# Removal of Artifacts from EEG Signals: A Review

**DOI:** 10.3390/s19050987

**Published:** 2019-02-26

**Authors:** Xiao Jiang, Gui-Bin Bian, Zean Tian

**Affiliations:** 1 Institute of Automation, Chinese Academy of Science, Beijing 100190, China; jx19950427@163.com; 2 School of Big Data and Information Engineering, Guizhou University, Guiyang 550025, China; tianzean@126.com

**Keywords:** electroencephalogram, artifact removal techniques, artifacts

## Abstract

Electroencephalogram (EEG) plays an important role in identifying brain activity and behavior. However, the recorded electrical activity always be contaminated with artifacts and then affect the analysis of EEG signal. Hence, it is essential to develop methods to effectively detect and extract the clean EEG data during encephalogram recordings. Several methods have been proposed to remove artifacts, but the research on artifact removal continues to be an open problem. This paper tends to review the current artifact removal of various contaminations. We first discuss the characteristics of EEG data and the types of different artifacts. Then, a general overview of the state-of-the-art methods and their detail analysis are presented. Lastly, a comparative analysis is provided for choosing a suitable methods according to particular application.

## 1. Introduction

With the emergence of non-invasive techniques, the research of neuroscience, cognitive science and cognitive psychology has been developed by electroencephalograph (EEG), functional near-infrared spectroscopy (fNIRS), magnetoencephalography (EMG) and other key tools [1]. EEG is one of the tools used for analyzing brain activity, which signals can be recorded from several electrodes on the scalp [2]. As a special and complicated biological electricity signal, it reflects the functional state of brain allied to the person’s mental condition, from which we can extract the vital information, and further monitor patient’s heath [3], diagnosis and identify different brain conditions [4,5]. However, EEG is high temporal resolution and its signals are easily contaminated by undesired noise, which will resulting in various artifacts [6]. The cause of artifacts may arise from measurement instrument and human subjects, the prior one including faulty electrodes, line noise and high electrode impedance [7], which can be avoided by more precise recording system and strictly recording procedures, whereas physiological artifacts are more complicated to remove. For instance, eye movements, eye blinks, cardiac activity and muscle activity occurred in EEG signal are some major types of physiological artifacts [8].

**Figure 1:**
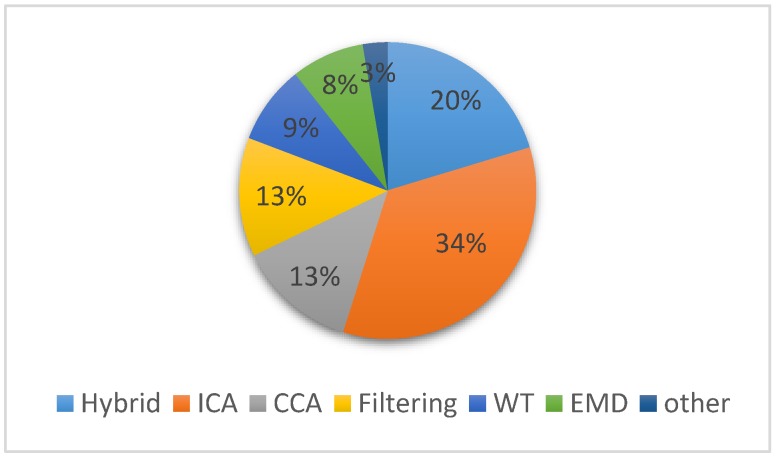
Percentage of the number of references published during the past three years.

Such physiological artifacts may interfere with neural information and even be used as normal phenomena to misleadingly drive a practical application such as brain-computer interface [9]. Furthermore, artifacts might also imitate cognitive or pathologic activity and therefore bias the visual interpretation and diagnosis in clinical research such as sleep order, Alzheimer’s disease [10,11], etc. Therefore, the requirement of artifacts identification and removal, either in clinical diagnosis or practical applications, are the most important preprocessing step prior to be utilized. One straightforward way is to apply precautions to avoid unnecessary motion incurring artifacts, but it will get into trouble if subjects have inability to follow such extra instruction, and furthermore this method is unwieldy either for clinical or household applications. Instead of artifact avoidance, manual segment rejection directly omit epochs that contaminated by artifacts. Consequently, this method will significantly loss possibly useful neural signals. To this end, a variety of efficient techniques for artifact removal, especially for physiological artifacts, has been proposed in the published literatures. Such published techniques advanced by improving existing algorithms, combining various methods or making removal process automatic can be primarily separated into two categories: either by estimation of the artifactual signals using reference channel or by decomposing the EEG signal into other domains. Alternatively, these techniques range from Regression [12], Blind Source Separation (BSS) [13,14,15], Empirical-mode Decomposition (EMD), Wavelet Transform algorithm to their hybrid methods [16]. To obtain a comprehensive overview of EEG artifact removal techniques developed in different studies, we used Google Scholar as the main search engine. The keywords were “electroencephalogram”, “EEG artifacts”, “denoising”, “artifact correction” and “artifact removal method”. Boolean operators (AND, OR, and NOT) were used to combine search terms. Figure 1 provides the chart that percentage of the number of literatures in the past five years. It indicate that the BSS-based methods, especially ICA, are the most commonly use algorithms. Also it is worthy to note that from the chart, due to the limits of single method like Regression and BSS, many researchers tend to prefer the hybrid method to enhance the performance of techniques in recent years. Despite the extensive research centered on artifact detection and removal of EEG signals reported in the literature to date, there is no consensus of one optimal solution for all types of artifacts.

Considering this issue, we attempt to do a comprehensive review of main methods proposed in literature for artifact removal on EEG. We firstly review the characteristic of EEG signal and the types of artifacts existed. Then, we present the widely applied removal techniques and their advantage and drawbacks. Finally, a comparative analysis according to particular requirement is discussed. We believe that the knowledge provided in this review can help to determine a removal technique, which satisfies the necessary requirements for a particular application.

## 2. Background

### 2.1. Characteristics of the EEG

EEG is the recording of the brain’s spontaneous electrical activity and provide the measurement Voltage fluctuations of brain activity [17,18]. The frequency of EEG signals range from 0.01 Hz to around 100 Hz, which can be divided into five frequency bands, and four basic types are summarized in Table 1.

### 2.2. Types of Artifacts

**Figure 2:**
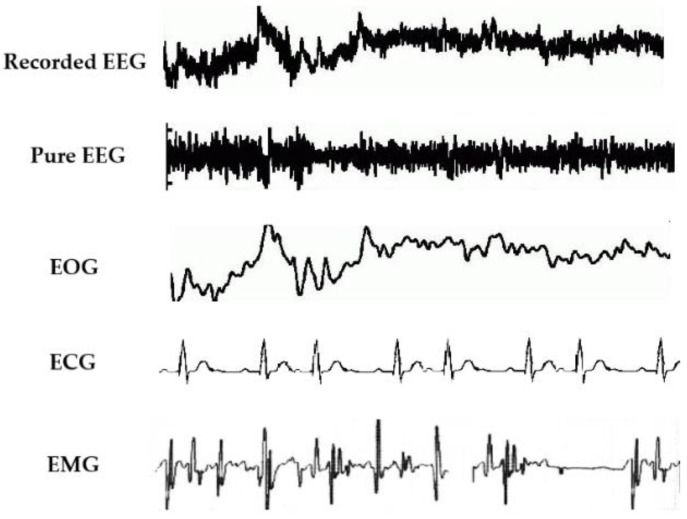
Physiological artifacts present in EEG signals.

Signal artifacts are more significant while collecting EEG data from recording systems [19,20]. These artifacts can contaminate the quality of EEG data. In this regard, a comprehensive knowledge of the types of artifacts is requisite to remove the artifacts or noise efficiently. Artifacts are unwanted signals which are mainly originated from environment noise, experimental error and physiological artifacts. Furthermore, the environment artifacts and experiment error, which come from external factors, are classified as extrinsic artifacts, whereas the physiological from body itself (e.g., eye blink, muscle activity, heart beat) can be categorized as intrinsic artifacts [21,22,23]. Figure 2 shows the three major physiological artifacts in the literature. The environment artifacts can be eliminated by a simple filter due to the frequency of such artifacts are inconsistent with desired signals [21]. Proper procedure and planning can reduced experimental error easily [19]. Nevertheless, the physiological artifacts are more difficult to be removed as they require particular algorithms [24]. We next discuss the major physiological artifacts affecting EEG data.

#### 2.2.1. Ocular Artifacts

Ocular artifacts generate significant artifacts in the EEG recordings [25]. The origin of ocular artifacts is eye movement and blinks which can propagate over the scalp and be recorded by EEG activity. More specifically, eye movement artifacts produce by changes in orientation of the retina and cornea dipole, and blink artifacts caused by ocular conductance due to the alterations of contact of the cornea with eyelid [26]. In addition, because of volume conduction effect, both ocular artifact and EEG activity propagated to head surface and record by the electrodes. Such ocular signals can be recorded using electrooculogram (EOG). The amplitude of EOG is generally many times greater than EEG [25,26,27] and its frequency are similar with the frequency of EEG signals. Worthy to note that not only EEG data can be contaminated by the EOG, but in turn, EOG can also be contaminated by EEG. Consequently, a bidirectional interference will introduce removal error when we remove EOG artifacts [28].

#### 2.2.2. Muscle Artifacts

Contamination of EEG data by muscle activity is a well-recognized tough problem as it arises from different type of muscle groups [27,29]. These artifacts can be caused by any muscle proximity to signal recording sites contraction and stretch, the subject talks, sniffs, swallows [21], etc. In theory, muscle artifacts measured by electromyogram (EMG) have a broad distribution from 0 Hz to >200 Hz [21,30]. The degree of muscle contraction and stretch will affect the amplitude and waveform of artifacts. It is extremely difficult to obtain the activity from a single channel measurement comparing to EOG and eye-tracking. Therefore, EMG artifacts are particularly challenging to eliminate. Additionally, EMG contamination and EEG have substantial statistical independence from each other both temporally and spatially. This imply that the Independent Components Analysis might be a suitable methods to remove EMG contamination [31,32].

#### 2.2.3. Cardiac artifacts

Cardiac artifacts can be introduced when the electrodes is placed on or near a blood vessel [29], in which the movement of expansion and contraction due to the heart. Such artifacts called pulse artifacts, whose frequency is around 1.2 Hz, can occur within EEG as a similar waveform, hence it is hard to remove [27]. Another cardiac activity known as ECG measure the electrical signal outputted from the heart [33]. In contrast to pulse artifacts, ECG can be measured with characteristic regular pattern, and be recorded aside cerebral activity, hence removing such artifacts may be easier just using a reference waveform.

#### 2.2.4. Extrinsic Artifacts

In addition to the artefacts mentioned above, external source of artifacts also have a damaging effect on EEG measurement. Instrument artifacts as a type of extrinsic artifacts originate from the electrode misplacement and cable movements. These artifacts can be removed by appropriate procedure and planning. The electromagnetic interference emitted from surroundings is another type external artifact that affect the EEG recordings. Such artifacts from environmental sources can be easily degraded by a simple filter due to its distinguishable frequency band. Despite the broad frequency band of white noise, a high-frequency filter still can remove majority of artifacts [13]. Since the activity of brain area can be observed in many channels, the coherence between EEG channels will introduce volume conduct artefact [34]. Relevant literature to deal with volume conduct artifact can be found in [35,36].

## 3. Single Artifacts Removal Techniques

### 3.1. Regression Methods

The traditional method for removing artifacts from EEG is the regression methods [37]. It is applied under the assumption that each channel is the cumulative sum of pure EEG data and a proportion of artifact [13]. Regression analysis first defines the amplitude relation between reference channel and EEG channel by transmission factors, and then subtracting the estimated artifacts from EEG. Thus, this algorithm requires exogenous reference channels (i.e., EOG, ECG) to omit different artifacts. When dealing with ocular activity, the EEG data can be obtained as:

EEG_cor_ = EEG_raw_ − γF(HEOG) − δF(VEOG) (1)

where γ and δ depend on the transmission coefficient between EOG and EEG, and ${\mathrm{EEG}}_{\mathrm{cor}}$ and ${\mathrm{EEG}}_{\mathrm{raw}}$ represent corrected EEG data and raw EEG data, respectively. HEOG and VEOG denote the recordings from horizontal and vertical EOG channels.

Hillyard and Gallambos [38] firstly proposed approaches based on time domain regression to remove ocular activity. Whitton et al. [39] introduced the frequency domain regression and combined this method with EEG detection software accordingly. However, either in time-domain or frequency-domain, such regression approaches are affected by bidirectional methods, [25]. This is due to ocular potentials contaminate EEG data, similarly the EEG data can contaminate ocular recording. To this end, Wallstrom et al. used a filtering prior to applying Bayesian adaptive regression splines [40,41]. In doing so, the issues of bidirectional contamination is substantially reduced.

Although the simplified model and reduced computational demands of regression methods, the need for one or more good regression reference channels limit their capacity for removing EOG and ECG [21]. Beyond that, anastomotic regression channels to eliminate each muscle artifact are not always available. Nevertheless, despite researches prefer blind source separation-based methods, regression-based algorithms are still the gold standard to assess the performance of new approach.

### 3.2. Wavelet Transform

Wavelet transform, transforming a time domain signal into time and frequency domain, has good time-frequency features relative to Fourier transform due to the better tunable time-frequency tradeoff and superiority of non-stationary signal analysis [42]. The transformation is accomplished by selecting the subsets of the scales ‘j’ and the time shift ‘k’ of the mother wavelet ψ(t). Mathematically:

(2) $$\psi_{j,k}\left(t\right)=2^{\frac{j}{2}}\psi (2^{j}t-k),$$

where j and k are integers. Then the wavelet transform can be performed by:

(3) $$W_{\psi }=<f,\;\psi_{j,k}>,$$

which means the inner product of time-domain signal and wavelet function.

The DWT (discreted wavelet transform) derived from continuous wavelet can be applied when the input signal, and the decomposition can be expressed as:

(4) $$X_{a,L}[n]=\sum_{k=1}^{N}X_{a-1,L}\left[2n-k\right]g\left[k\right],$$

(5) $$X_{a,H}[n]=\overset{N}{\underset{k=1}{\sum }}X_{a-1,L}\left[2n-k\right]h\left[k\right],$$

where g[n] is the low pass filter to generate low frequency component and h[n] is high pass filter to get high frequency component. After the decomposition of EEG data using wavelet transformation, thresholding is applied to discard the signal that contain artifacts. The remaining details are added up to reconstruct the clean signal [43]. Even though its versatility of artifact attenuation, the DWT fails to identify artifacts completely that overlap with the spectral properties, hence recent work prefer the combination of DWT with other methods, like ICA.

### 3.3. BSS

The BSS method includes a variety of unsupervised learning algorithms without prior information and extra reference channels. The general methodology of BSS can be described as follows. Let X be observed signals obtained from scalp electrodes. Also, let S be the source signals which includes original signals and artifacts. These source signals are linear mixed by an unknown matrix A:

(6) X=AS,

to get the observed signals. The BSS algorithm is a reversed version:

(7) U=WX,

where U is the estimation of sources and the W is the reverse mixing of X. Then components representing the artifacts are removed and the remaining components reconstruct EEG data to achieve the purpose of denoising. In that following, we describe some representative works that have adopted the BSS algorithms.

#### 3.3.1. Principal Component Analysis

PCA is one of the simplest and widely used BSS techniques, which algorithm is based on Eigen values of covariance matrix [44]. In this method, it firstly converts correlated variables into uncorrelated variables using orthogonal transformation. Such uncorrelated variables are called principal components (PCs). These PCs of EEG signals will be implemented using Single Value Decomposition (SVD).

Berg and Scherg [45] firstly introduced principal component analysis to remove ocular artifacts. In this work, the major components representing blinks and eye movements are extracted by PCA of variance. Then the artifact-free EEG data was obtained by rejecting the related components through an inverse operation. Casarotto et al. [46] demonstrated that PCA perform more computationally efficient than linear regression methods. However, the requirement that artifact components are uncorrelated with EEG data is generally hard to satisfy. Besides of that, PCA fails to separate the interferences when the potential of drifts and EEG data are similar. Consequently, the subsequent research prefer other flexible methods such as ICA.

#### 3.3.2. Independent Component Analysis

Another method known as ICA, assuming that signal sources are instantaneously linear mixtures of cerebral and artifactual sources, can decompose observed signal into independent components (ICs) [14]. Once ICs are extracted from original signals, the clean signal reconstructed by discarding ICs contained artifacts.

Considering that the simple network algorithm method achieved by Bell and Sejnowski blindly separate signals using information maximization, Makeig et al. firstly applied the ICA algorithm to analyze EEG and EPR signals [47]. In contrast to traditional artifact cancelling approaches, Vigaro et al. tested the ICA method on simulated and experimental data, and showed good performance in the separation of signals from their linear mixtures and extraction of the eye information present in EOG signals [48,49]. In 2000, Jung et al. removed artifacts from EEG by the extended ICA, and results comparing effectively to regression algorithm [50]. Romero et al. applied ICA to reduce EEG artifacts in different sleep stages, and found the bidirectional property of EEG and EOG had little effect on ICA [51]. A method using probability and kurtosis to eliminate semi-automatically was discussed by Delorme et al. [52]. In order to avoid the errors introduced by manually select components, Joyce et al. [53] introduced an automatic extraction and removal of eye movement artifacts after the ICA analysis. In recent years, the study on automatic removal of artifact based on ICA has been developed by [54,55,56,57,58]. Frølich and Dowding compared five commonly used variants of ICA methods suitable for oscillatory activity. And the author find that the adequately high-pass filtering is very important and the extended Infomax perform best [59].

As an extension of PCA which be constrained to transform directions that are orthogonal, ICA has been proven more effective and flexible in source separation of EEG signals form artifacts under the premise below:

(1) Source signals are statistically independent from each other and instantaneously mixed.
(2) The dimension of observation signal must be greater than or equal to source signal [25,60].
(3) Sources are non-Gaussian or only one source are Gaussian.

In general, all artifacts like eye movements, eye blinks, cardio activities are generated by mutually independent source and volume conduction is linear, which makes the first premise is reasonable. However, the acquisition of biomedical signal is usually not linear instantaneous, and therefore, a Convolutive ICA (CICA) approach considering weighted and delayed contributions of signals was proposed in [61]. Additionally, ICA can estimate original signals which are non-Gaussian, but unfortunately sources are usually not known to be Gaussian or non-Gaussian. Another problem is that the scalp EEG are the result of the joint activity from multiple neurons, therefore, separating the source signals when the number of sources is greater than the number of sensors is promising.

#### 3.3.3. Canonical Correlation Analysis

Canonical correlation analysis (CCA) is another commonly employed BSS technique. Unlike ICA method that use higher order statistics, CCA use second order statistics which bring shorter computational time. CCA also differ from ICA in its conditions to separate components. The CCA separate components from uncorrelated sources whereas ICA from statistical independent sources [62]. CCA find the linear relation between two multi-dimensional random variables by maximize the pairwise correlations across the two data sets. [63]. In contrast to ICA that only takes statistical distribution of the same sample values into account, CCA consider the autocorrelation in the source signals and demonstrated that have similar qualitative results, but consume a little computational complexity. The CCA has been applied to remove muscle artifacts from EEG signals has been first discussed in [64]. CCA has also been used in the removal of muscle artifacts from EEG recordings of spoken language production [65]. The author investigated the difference in autocorrelation between brain and muscle artifacts. Then the components with least autocorrelation were selected and removed. In order to further explore potential nonlinear process, an eigenspace maximal information canonical correlation analysis (emiCCA) framework was proposed in [65]. Due to the form of muscle activity, which do not have stereotyped topography, the CCA algorithms outperform ICA to remove muscle artifacts.

#### 3.3.4. Source Imaging Based Method

EEG source imaging (ESI) is a model-based imaging technique that combines temporal and spatial components of EEG to find the source of scalp-recorded potentials [66]. There are two fundamental issues in ESI: forward and inverse problem. The equivalent distributed dipole layer inversion from scalp recordings is a linear inversion that can be solved by minimum norm (MN). In current practice, various regularizations or weighted MN solutions are practically utilized [67]. Then the equivalent distributed source from EEG recordings can be reconstructed. Next, the first component discarded after the analysis of PCA, remaining components can be reconstructed the artifact-free EEG [68,69].

### 3.4. Empirical Mode Decomposition

Empirical mode decomposition (EMD) was first discussed in 1998 [70] as a heuristic technique for non-stationary and non-linear signal processing. EMD algorithm decomposes the signal, x[n], into a set of components with amplitude-frequency modulated, b[n], called intrinsic mode functions (IMFs) [71,72]. In the whole data set and all point, every IMF must be satisfies that the number of extrema are same with the number of zero crossings or differ at most by one, and the mean value of the envelope defined by the maxima and minima must be zero. Therefore, the EMD technique is empirical and data driven technique, whereas other methods depend on the selections of basic functions, such as wavelet analysis [73]. A sifting procedure is taken to calculate the IMF of a given signals and the steps shown below:

(1) Set b[n] equal to input signal sequence x[n].
(2) Calculate all the local maxima and local minima, and connect them separately with cubic spline interpolation. The upper envelope u[n] and lower envelop l[n] are obtained.
(3) Calculate the mean value as: μ[n] = (u[n] + l[n])/2, and subtract it form original.
(4) Decide whether b[n] is an IMF or not according to the condition described above.
(5) Repeat steps 2–4 process until an IMF is obtained and assign b[n] to b_k_[n].
(6) Once a IMF is obtained, generate the residue r[n] as: r[n] = r[n] − b_k_[n].
(7) Repeat steps 1–5 on the residue as the input signal sequence until the final residue is a constant, a monotonic function, or a function with only one maximum and one minimum.

Then the original signal can he reconstructed by:

(8) $$x\left[n\right]=\sum_{k=1}^{m}b_{k}\left(n\right)+r\left(n\right),$$

where r(n) represent the final residue signal [74]. Once the IMF determined, the artifact components of EEG data can be reflected, and then selected and removed. Finally, the pure EEG signal can be reconstructed by the newly IMFs. One drawback of EMD algorithm is that the sensitivity of noise, which incurring mode mixing complications [75]. The details about enhanced-EMD (EEMD) algorithm are discussed in [76], in which an average of a number of ensembles of EMD was utilized as the optimal IMFs, as a result, the robustness of EMD was improved. In some case, the reconstructed remaining IMFs can be entered to an extra artifact removal environment to enhance the quality of EEG data [77]. Another modified EMD, MEMD, also be proposed to simultaneously decompose multivariate signals into multivariate IMFs. Due to the simultaneously analysis of intrinsic modes across multiple channel, MEMD can more efficiently and accurately to remove artifacts, especially for broadband muscle artifacts [78].

### 3.5. Filtering Methods

Numerous filtering methods was employed in the cancelation of artifacts from the EEG, for instance, adaptive filtering, wiener filtering and Bayes filtering, in which different methods implemented with different principle of optimization [79]. Nevertheless, for the intention to minimize the mean square error between the predicted EEG and primary EEG, a weighting coefficient W will be adapted. Following article briefly illustrates two commonly used filtering approaches accordingly.

#### 3.5.1. Adaptive Filtering

**Figure 3:**
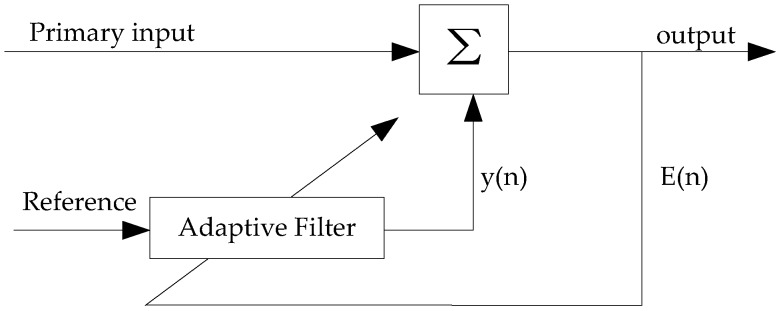
Functional diagram of an adaptive filter system.

The underlying mechanism of adaptive filtering is to quantize the amount of artifactual contamination in the primary input, by iteratively adjusting the weights according the optimization algorithm, and subtract it from EEG with artifacts signals [80]. An illustration of the adaptive filtering is presented in Figure 3. The primary input is modelled as a mixture of clean and pure EEG data and an artifact source with the formula:

(9) $${\mathrm{EEG}}_{\mathrm{pri}}(n)={\mathrm{EEG}}_{\mathrm{pure}}\left(n)+N(n\right),$$

where ${\mathrm{EEG}}_{\mathrm{pri}}$ and ${\mathrm{EEG}}_{\mathrm{pure}}$ represent the primary signal and desired signal, respectively, and N represent noise signal which is an EOG artifact or an ECG interference according to the artifact to remove. Reference channel is given as one of the input to the filter. In order to obtain the pure signal, ${\mathrm{EEG}}_{\mathrm{pure}}$, optimization algorithm like least mean squares (LMS) is used to help adaptive filter to upgrade its weight parameter. Another alternative optimization algorithm is recursive least squares algorithm (RLMS) which convergence faster than LMS, but still requiring high calculation cost [81]. Disadvantages are that additional sensors is needed to provide reference inputs [82].

#### 3.5.2. Wiener Filtering

The wiener filtering is also an optimal filtering as the adaptive filtering, however, wiener filter technique is a linear statistical filtering technique used to estimating the true EEG data with the purpose to develop a linear time invariant filter to minimize the mean square error between the pure EEG data and the estimated signal [83]. The linear filter is found by estimating the power spectral densities of the measured signal and the artifact signal, since there is no prior knowledge on the statistics [84]. Although wiener filtering eliminate the limitation of extra reference, but the requirement of calibration will add the complexity of its application.

### 3.6. Sparse Decomposition Methods

Sparse component analysis (SCA) is another effect signal processing method to decompose signals sparsely in over-completer dictionary [85]. The over-complete dictionary can be calculate from complete dictionary by over-sampling. A dictionary can be constructed by waveforms or atoms, such as wavelet, Fourier and Dirac basis. After over-sampling, the orthogonality may not be true whereas the basis in the complete dictionary is orthogonal to each other. Signal sparsity can be measured with *l_p_* (0 ≤ *p* ≤ 1) as follows:

(10) $${\left\|x\right\|}_{p}={\left(\overset{n}{\underset{j=1}{\sum }}{\left|x_{j}\right|}^{p}\right)}^{\frac{1}{p}},$$

where X represents the signal, and n is the dimension of X [86]. Mallat and Zhang introduced a matching pursuit (MP) algorithm that decompose a signal into sparse representation [87]. The sparse decomposition of signal corresponding dictionary is obtained after several iterations, and each iteration choose a certain waveform with maximum inner product between itself and residual signal as the optimal one. A matching pursuit can isolate the signal structures that coherent with respect to a given dictionary. A modified MP method termed two dictionaries MP (TDMP) was proposed in to decompose signal more sparsely, and the decomposition results certify that TDMP are superior to MP [88]. In EEG analysis, Li et al. combined L_1_ norm-based Eigen decomposition into Common Spatial Patterns, and it effectively improve the robustness of BCI system to EEG outliers [89]. Another work developed a novel auto-regressive object function constructed in Lp norm space to compress the artifacts on EEG analysis [90].

## 4. Hybrid Methods

Beside from the algorithms concluded above, there are still many efficient and innovative methods. To exploit the advantage of each method, recently, several researchers have opted to use a hybrid strategy, which is a combination of two or more methods. Some major hybrid methods are discussed below.

### 4.1. EMD-BSS

**Figure 4:**
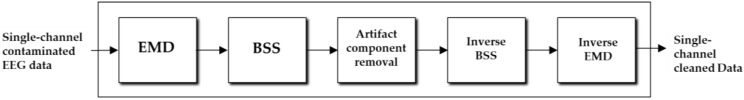
Process flow of EMD-BSS method.

In conjunction with ICA, these intrinsic mode functions (IMFs) are decomposed by an EMD or EEMD algorithm. Then these IMFs be passed as inputs to an ICA algorithm with the goal of estimated the source signals. Sequentially, reconstructing the new IMFs through multiplication between mixing matrix and IC components extracted without artifact, and the original signal can be obtained by summing remaining IMFs [91]. Figure 4 shows the general schematic of EMD-BSS method. The study of EEMD combined with ICA to remove EMG and ocular artifact from EEG was first present in [79]. The author also compared this algorithm with single-channel ICA and wavelet-ICA on real EEG signals. The results show that the EEMD-ICA algorithm has the best performance. Similar manner to EEMD-ICA, the EEMD-CCA [92,93,94,95], remove contaminated IMFs and then using unmixing matrix W, determined by CCA algorithm, to reconstruct source signals [96]. Another method utilized interchannel dependence information seen in few channel situation by combining multivariate empirical mode decomposition (MEMD) and CCA was discussed in [78]. Although it has been proven that MEMD-CCA is one of the most promising tool for muscle artifact removal under few-channel scenarios, the heavy computational cost limits it to be used in offline situation.

### 4.2. Wavelet-BSS

**Figure 5:**
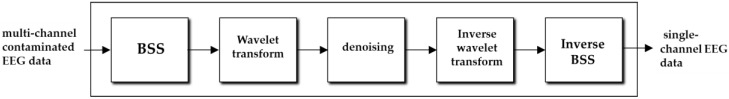
Process flow of BSS-WT method.

ICA has crucial limitation that the number of sources need to be equal to the measurement sources, whilst WT fails to work when the artifacts overlap in the spectral domain, thus, a wavelet-ICA technique has been proposed in [97,98,99,100] to combine the positive portion to avoid shortcomings. Lin et al. [97] used WICA for single-channel artifact removal and Winkler et al. [99] removed contaminated EMG signals from EEG data. Firstly, the recorded EEG data is decomposed by wavelet transform and then the wavelet resolution contained probably artifactual components are fed into a chosen ICA algorithm. Finally the selecting components corrupted with artifacts removed and reconstruction of artifact-free EEG signals are performed using preserved wavelet components and disposed components. Figure 5 illustrates the process flow of Wavelet-BSS algorithms. Another commonly used version reversing the order of wavelet transform and ICA or CCA algorithm has been discussed in [101,102,103,104]. Other than ICA or CCA, wavelet can also be used with together PCA, as presented in the work of Kevric and Subasi [105,106]. In their method, wavelet analysis decompose the EEG signals into different frequency bands and then PCA is applied to obtain new coefficients of bands [107]. Conventional linear artifact removal methods usually smooth out the rapid jitter in the signal. The nonlinear filtering techniques Multiscale Principal Component Analysis (MSPCA) will preserve substantial amount of changes in the recordings and provide enhanced artifact removal performance than sole PCA algorithm [108].

### 4.3. BSS and Support Vector Machine

**Figure 6:**
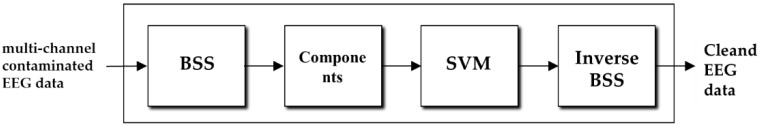
Process flow of BSS-SVM method.

Another hybrid method to extend the use of BSS is BSS-SVM. First, the recorded EEG data are decomposed into multiple components using BSS algorithms. Next, several features of components like temporal, spatial and statistical features are extracted. Then the features are used as input to a set of linear SVM classifier to identify artifact components. Finally, the remaining components are used to reconstructed artifact-free signals [21]. Figure 6 show the schematic of BSS-SVM method. Shoker et al. [109] first fused BBS and SVM to remove the eye-blinking artifacts. Halder et al. re-introduced this approach to classifier the artifactual components in EOG and EMG recording [110].

## 5. Comparative Analysis

The methods discussed above are among the most commonly used in EEG artifact removal. Some of these methods dealing with artifacts seed to minimize their presence by restricting eye movements and blinking during data collection or by excluding artifact-contaminated trials from the analyzed data.

Other methods seek to correct for ocular artifacts in the data, including regression in the time or frequency domain. Besides, others blindly separate artifacts from EEG signals. A detailed comparison between the mentioned techniques is provided in Table 2. There are a number of factors affect the choice of which algorithm to employ, which we discussed later.

Most of the EEG-based practical applications are often required real-time signal processing and can be robust to artifacts. This required the artifact removal methods implemented are capable of automatic and low computational cost. Automatic process means the chosen method can automatically identify and eliminate the artifact components without manual intervention. Regression and filtering approach can execute automatically when they have a reference signal. Moreover, BSS methods will be automatic when there is a subsequent procedure, as mentioned above, like SVM. Among these BSS methods, although ICA are the most commonly used techniques, the disregard of temporal or spatial relations within sources will result the loss of relevant information. But CCA algorithm can solve this problem and have a little computational time, which makes the algorithm applicable for real-time performance. Another factor which should be taken in account is the number of record channels. Especially for the home healthcare environment, the less of channel are often expected. BBS algorithms cannot be utilized in such situation, due to the principle of BSS that more channels will bring better accurate. But wavelet transform and EMD-based methods can executed with single channel since it can decompose a single records into multiple components [111]. However the reduction of measurement channel will cause the increase of computational complexity which will not be suitable for BCI and neurofeedback (NF) applications. In addition, the automatic methods are not usually general for artifact removal since there are multiple types of artifacts that are existed in the recordings. Hence the availability of reference signals will improve the accuracy and robustness of artifact removal by provide satisfactory complementary information. Also the information of the epochs of artifacts obtained by reference channel will reduce the computational cost. But the reference channel of each muscle contributing to EEG muscle artifacts are not feasible.

Apart from methods commented above, there are plenty of innovative and efficient approaches for artifact removal have been recently proposed. For instance, Corradino et al. [112] combined ICA with regression to automatically check, detection and clean residual artifacts. The technique presented to clean ICs can avoid the cut-band solutions. Recently, applied a multinomial regression classifier to establish a multiclass artifact identification system, which can automatically selected frequency, spatial and temporal features of the ICs. Considering that the trend of healthcare systems are developed toward few channels, Davies and James explored a single channel ICA method to decompose single channel signal into independent sources [113]. Another study developed the SASICA software plugin for the EEGLAB toolbox that encapsulating several artifact selection approach [114]. The interactive plots produced by this software guide human user to decide the removal of artifactual ICs. Daly et al. [115] proposed a method that combined wavelet decomposition, ICA and thresholding termed as FORCe. This online artifact removal method use for BCI applications. In 2016, a new method for real-time detection of eyeblink artifacts using an automatic thresholding algorithm was introduced [116]. Zou et al. extended the ICA method by the hierarchal clustering of IC features, which can identify both physiological and non-physiological artifacts form the EEG signals of BCI applications [117]. A recently emerging BSS algorithm which integrating the advantages of CCA and ICA into one single framework, independent vector analysis (IVA), remove muscle artifact by synchronously extracted the sources with maximal independence and maximal autocorrelation [118,119]. In addition, the combination of EEMD and IVA have been demonstrated that MEMD-IVA outperform other existing methods in a few channel situation [75]. More recently, a modified joint blind source separation (JBSS) approach and quadrature regression IVA (q-IVA) provide a more effective artifact removal technique in both time and frequency domain, paving the way for future research [120]. In addition, a filter-bank based artifacts removal approach was proposed by Dhindsa [121]. This method uses a supervise machine learning to detect artifacts by single channel, and outperforms Fully Automated Statistical Thresholding for EEG artifact Rejection (FASTER) due to its ability to identify small artifacts in the presence of high amplitude EEG. Mohammadpour et al. [122] made use of a Hidden Markov Model (HMM) machining learning method to remove eyeblink artifacts. Contrary to conventional algorithms, such machine learning based approaches that identify artifacts using huge datasets are tending to be a new research focus.

To conclude, ICA-based algorithms can deal with all kinds of artifact occurred in EEG recordings. Regression and adaptive filter are more feasible choices when the reference channels for specific artifacts are available. Apart from ICA, CCA and its combinations of other methods seem to be a good choice for removal of muscle artifacts. For application to a few channels, EMD, IVA, and its hybrid methods with BSS or WT could be an ideal choice. However, the requirement of a reference signal limits adaptive filter or regression methods to the removal of particular types of artifact. Wavelet transform fails to identify completely artifacts that overlap with spectral properties. EMD also suffers from the drawback of mode-mixing. Therefore, it is quite difficult to find a single method that is both efficient and accurate enough to satisfy all the conditions perfectly.

## 6. Conclusions

EEG signals are generated from the cerebral cortex and always be contaminated by some disturbances. In spite of the fact a number of techniques have been developed for removing undesired artifacts, an artifact removal method that combines high accuracy and algorithmic efficiency still needs to be identified. This paper summarized the primary techniques based on conclusions made in the published literatures. The advantages and the drawbacks of each method are also highlighted. Although most of the removal algorithms offer good performance, the methods listed above suffer from different limitations when utilized in a particular EEG-based application. Indeed, some methods are only focused on the detection and removal of particular artifacts, such as EOG, ECG, EMG. Some methods need reference channels to enhance the accuracy of artifact removal, which is not feasible of some specific applications. Some methods like BSS or Wavelet remove artifacts with great accuracy, however, methods operating with high computational complexity may not be suitable for online applications. Therefore, there is no optimal choice for remove all types of artifacts. Thus, one of the future objectives of the effective attenuation of artifacts is to develop an application-specific algorithm with better time and accuracy efficiency. Also in the current trend of artifacts removal, it can be concluded that the future directions will combine machine learning and traditional approaches for effective automatic artifact removal. Apart from that, new artifact removal algorithms for numerous types of artifacts in the multiple scenarios still need to be identified.

## Figures and Tables

**Table 1:** Basic brain wave with their frequency.

Band Name	Frequency (Hz)	Interpretation
Delta	<4	Deep sleep
Theta	4–8	Relaxed state and meditation
Alpha	8–13	Relaxed state of consciousness
Beta	13–30	active thinking

**Table 2:** Comparative analysis of methods mentioned above.

Method	Additional Reference	Automatic	Online	Can Perform on Single Channel
Regression	Y	Y	N	N
Wavelet	N	Y	N	Y
ICA	N	N	Y	N
CCA	N	N	Y	N
Adaptive filter	Y	Y	Y	Y
Winner filter	N	Y	N	Y
Wavelet BSS	N	N	N	Y
EMD BSS	N	N	N	Y
BSS-SVM	N	Y	Y	N
